# Nurses joining family doctors in primary care practices: perceptions of patients with multimorbidity

**DOI:** 10.1186/1471-2296-11-84

**Published:** 2010-11-04

**Authors:** Martin Fortin, Catherine Hudon, Frances Gallagher, Antoine L Ntetu, Danielle Maltais, Hassan Soubhi

**Affiliations:** 1 Department of Family Medicine, Université de Sherbrooke, Sherbrooke, Canada; 2 School of Nursing Sciences, Université de Sherbrooke, Sherbrooke, Canada; 3 Department of Humanities, Université du Québec à Chicoutimi, Saguenay, Canada

## Abstract

**Background:**

Among the strategies used to reform primary care, the participation of nurses in primary care practices appears to offer a promising avenue to better meet the needs of vulnerable patients. The present study explores the perceptions and expectations of patients with multimorbidity regarding nurses' presence in primary care practices.

**Methods:**

18 primary (health) care patients with multimorbidity participated in semi-directed interviews, in order to explore their perceptions and expectations in regard to the involvement of nurses in primary care practices. Interviews were audio-recorded and transcribed. After reviewing the transcripts, the principal investigator and research assistants performed thematic analysis independently and reached consensus on the retained themes.

**Results:**

Patients with multimorbidity were open to the participation of nurses in primary care practices. They expected greater accessibility, for both themselves and for new patients. However, the issue of shared roles between nurses and doctors was a source of concern. Many patients held the traditional view of the nurse's role as an assistant to the doctor in his or her various duties. In general, participants said they were confident about nurses' competency but expressed concern about nurses performing certain acts that their doctor used to, notwithstanding a close collaboration between the two professionals.

**Conclusion:**

Patients with multimorbidity are open to the involvement of nurses in primary care practices. However, they expect this participation to be established using clear definitions of professional roles and fields of practice.

## Background

In primary care, a high number of patients present multimorbidity [1,2], defined as the simultaneous presence of two or more medical conditions in the same patient [3]. Many studies have shown that multimorbidity was associated with negative outcomes [4-17]. A number of problems in following up such patients have been identified, such as increased costs [18-20], difficulty in maintaining continuity of care [21] and compliance [22,23], greater use of emergency services [24], difficulty in observances and fragmentation of care [24,25], conflicts between interventions [24], difficulty in applying guidelines [26,27]. In the presence of multimorbidity, providing care to patients becomes much more complicated as in cases where focus on a single disease can lead to inadequate attention given to other conditions present in one patient, or in the case of the treatment of one disease adversely affecting another [28].

Nevertheless, this complicated situation, along with the shortage of family physicians in Canada, offers a unique opportunity to develop innovative forms of service delivery in this context to better meet the needs of vulnerable patients. Among the strategies used to reform the primary care system, the integration of nurses into family practices appears to offer a promising avenue, and is included in health care models currently in use. Studies and practice models across Canada and in other countries report increased provider and patient satisfaction, access to care and decreased hospitalization [29]. In the province of Quebec, one model is the *Groupes de Médecine de Famille *(Family Medicine Groups: FMG). An FMG is an organization that offers primary care services to rostered individuals. One of its approaches to ensure better accessibility to services is the integration of nurses with family doctors [30,31]. Working within such a structure requires a redefinition of the doctor-nurse partnership [32]. Although there is extensive literature addressing nurses being involved in primary care practices [32-39], very few studies have considered patients' perceptions on this addition [40,41]. If we are to build a healthcare system that is truly patient-centered, we must give voice to patients in regards to what they expect from that system [42]. The transformation of traditional family practices in Québec has been the subject of much discussion and attention in the media. This study took place at the beginning of this transformation and aimed to explore the perceptions and expectations of patients with multimorbidity regarding the presence of nurses in primary care practices.

## Methods

A qualitative descriptive clinical study was conducted using semi-structured interviews adapted from the pragmatic method described by Crabtree and Miller [43,44]. Combining perspectives of applied anthropology and primary care clinical practice, clinical research elicits patients' viewpoints to better inform practice [44]. To inform the presence of nurses in primary care practices, we were interested in capturing how patients perceive the idea of having a nurse join their family doctor's practice and participate in the patient's follow-up. This study is also inspired by "qualitative description" as Sandelowski described it [45,46]. The study was conducted in the Saguenay region of the province of Quebec (Canada). Sampling was purposeful with variation [43] regarding the model of care experienced by the patient. In fact, patients were recruited from the practice of six family doctors working in different settings representative of the Quebec primary healthcare system: a Family Medicine Group (described above), a CLSC (*Centre Local de Services Communautaires */Local Community Services Centre: health organization that provides primary health care, health promotion and community development services) and a Family Medicine Unit (FMU: a primary health care clinic affiliated with a University), where patients have access to follow-up care by doctors and registered nurses, as well as a private clinic, where follow-up is primarily ensured by a doctor. In accordance with this choice of settings, we were interested in patients with various experiences with nursing practices. Therefore, we also purposefully included patients without any experience with nurses in primary care. It is important to mention that nurse practitioner in primary care was not an acknowledged profession in Quebec at the time of this study as opposed to other Canadian provinces. Nurses had college or university (undergrad) level training in these settings.

In order to select patients with a high exposure to the health care system, we recruited those with five or more chronic diseases, as listed in their patient file. Our previous data show that this type of patient accounts for approximately 50% of family doctors' practices [1]. Patients presenting cognitive disorders, unstable states (medical or psychiatric), or serious difficulties understanding or expressing themselves were not included in the study. Thus, we limited our observations to patients that were fairly typical of primary care and excluded those who were extremely vulnerable. We assumed that by recruiting between 15 and 20 patients, we would reach data saturation [43]. The project was approved by the ethics committee of the *Centre de santé et de services sociaux de Chicoutimi *(CSSSC).

### Interview guide

Two members of the research team developed an interview guide based on our review of the literature on FMGs and the role of nurses in primary care settings and submitted it to the entire team for review. It is reflective of, and strengthened by, perspectives from family medicine, nursing and social work disciplines.

Open-ended interview questions covered: continuous follow-up with a family doctor, membership in an FMG, changes in services received from an FMG, continuous follow-up with a nurse in same care setting, collaboration in care (defined very broadly during the interview as sustained follow-up care involving at least one family doctor and one nurse in primary care over a care period). Participants were also asked to reflect on issues regarding their expectations and concerns about family doctors and nurses in primary care, the advantages and disadvantages of family doctors and nurses working collaboratively for the delivery of their care, problems or conditions they are comfortable having managed or discussed or trust with doctor/nurse and ideal care scenarios involving nurses and family doctors.

### Process

Participants were recruited in collaboration with their family doctor who provided the researchers with a list of patients that fit the inclusion criteria. After providing informed consent, participants were invited to complete a sociodemographic questionnaire and to participate in a one-hour interview. At the beginning of the interview, participants were asked to talk about their health problems and the primary care they had received for these problems. The discussion was then directed towards their experience with the professionals who provided the primary care focusing on family doctors and nurses. Patients' perception of the presence of nurses in family practice related to both how they experienced it and their anticipation of such experiences. Participants described their past or current experiences regarding nurses working with doctors in primary care, and those with no experience were encouraged to talk about their perception of this type of practice for their health care in the future. Next, the discussion included ideal scenarios of primary health care provision. Interviewers used the guide as a flexible and evolutive tool so they could explore other topics that arose during the interview. Interviewers were required to explain the context of doctors and nurses working within an FMG to the participant when presenting the consent form prior to the interview, since some participants had no experience with this type of healthcare setting. Interviews were held at the Family Medicine Unit of the *Centre de santé et de services sociaux de Chicoutimi *(CSSSC) or at the patient's home. All interviews were completed in less than 60 minutes and were recorded and transcribed verbatim. The first four interviews were observed through a one-way mirror by a team of researchers and family medicine residents for further clarification of the interview guide and to complete the research assistant's training. The research assistant, a qualified nurse, conducted the following interviews from April to July 2006, while remaining in constant communication with the principal investigator, who provided continuous feedback. The assistant was instructed on the importance of neutrality, objectivity and attentive listening.

### Analyses

Descriptive analyses of the sample were conducted using the data obtained from the sociodemographic questionnaires. Transcripts were validated with the interviewer's field notes and with the original recordings and reviewed by the research assistant and principal investigator. NVivo was used to help with data coding. An initial analysis schema with a minimal list of pre-established codes was elaborated based on our literature review and analysis of the first interviews. As part of a semi-inductive analysis process [47], the principal investigator and the research assistant immersed themselves in the data and made several readings. Categories and themes that emerged were added to the schema. Cases summaries were then constructed and cross-case analysis was done through the construction of a conceptual matrix as suggested by Miles and Huberman [47]. This allowed us to group patients according to common experiences, characteristics and patterns. The principal investigator and research assistants performed independent analyses of all interview observations. Results were pooled and compared by a third researcher and showed excellent convergence. Consensus was reached on the retained themes after discussion with team members from various disciplines (family medicine, nursing, social work) to strengthen the analysis.

## Results

Eighteen interviews were conducted. Table 1 presents participant demographic characteristics. The chronic conditions listed in patients' files are numerous and varied, including hypertension, hyperlipidemia, diabetes, osteoporosis, osteoarthritis, cardiovascular heart disease, heart failure, peripheral vascular disease, chronic obstructive pulmonary disease, asthma, migraine, depression and other conditions frequently seen in primary care, in various combinations. As planned, we were able to verify from the interviews that patients had various levels of experience with nurses working with doctors and from those with experience (n = 15), levels varied from low to high. An example of low level of experience (coded L with the participant number in quotes below) was a few encounters with a nurse and a doctor during a limited time for initial training on diabetes control over a several-week period. An example of high experience (coded H with the participant number in quotes) was a regular follow-up by a nurse and a doctor within the context of an FMG with repeated visits to both professionals specifically. Some experiences were outside the context of primary care (e.g. follow-up by a nurse (home visits) and family doctor (office visits) following surgery with a specialist). Nevertheless, we were able to classify all participants regarding their openness to the involvement of nurses in primary care practices (through the exploration of ideal scenarios when participants did not relate any experience) and found the majority to be receptive (13/18).

**Table 1:** Characteristics of the sample

Characteristic	Interviews completed (n = 18)
Mean age, (SD): yr	63.8 (7.9)
Male, %	38.9
Education level, %	
<8 y	22.2
8 to 12 y	38.9
Post-secondary (college or university)	38.9
Household income in Canadian dollars, %	
<$10,000	5.6
$10,000-$29,999	50.0
$30,000-$49,999	22.2
≥$50,000	22.2
Marital status, %	
Married	61.1
Divorced/Separated	11.1
Widowed	22.2
Single	5.6

Three major themes emerged from the analysis and are summarized in Table 2. Themes are reviewed in detail below and illustrated with quotes from the interviews.

**Table 2:** Summary of analyses

Themes	Sub-Themes
**Expectations of improved primary care practices**	Accessibility
	Continuity
	Better follow-up
	Complementarity
	Fear of limited access to one's doctor
	
**Dual views of the nurse's role and competency**	Traditional nurse's role
	Broader nurse's role
	Nurse's competency questioned
	
**Conditions for the successful involvement of nurses in primary care practices**	Nurse-doctor communication
	Demonstrated nurse's expertise
	Continuous medical education
	Stability of professionals
	Use of care protocols by nurses

### Expectations of improved primary care practices

Main expectations expressed were about accessibility and continuity of care. Many felt that the participation of nurses in primary care practices could improve accessibility to care for themselves as well as for others. Being able to reach the family doctor or the nurse in a timely manner when required meant that their care needs would be effectively responded to within an acceptable timeframe. Because the nurse was perceived as being able to facilitate contact with the doctor, patients generally expected that their doctor would be more readily accessible for themselves or for other patients. In addition, patients expected greater continuity of care and a higher quality of medical follow-up, simply through contact with both the nurse and the doctor instead of a single professional.

Participant 10-H: "I really know that if I need to, for an emergency, I can see a nurse, who would see that my case was serious! [...]You always feel more secure when you know that you can call, when you know that a nurse is there to talk to, or that she'll come and see you right away, if it's an emergency, [...] it could allow you to see the doctor more quickly."

Participant 11-H: "[...] if it's the nurse that's doing it, the doctor can stay longer and see other patients that are waiting [...] This lets the doctor see other people that haven't been able to find a family doctor, maybe simply because the doctor doesn't have enough time [...]."

Participant 13-L: "Well, I'm going to be treated faster, because if there's anything that the nurse can do, it's going to be done right away; the nurse is going to handle it, and he (the doctor) can see another patient."

Patients expressed one main concern that contradicts the above statements: the fear of not being able to see their family doctor. The possibility of having a follow-up visit with the nurse instead of the doctor raised feelings of hesitation and insecurity.

Participant 4-L: "Meeting with my own doctor! I feel that ... it seems to me (hesitation), of course I would feel more secure with my doctor than with the nurse! Anyway (hesitation), you can meet with the nurse, but ... replacing the appointment with, ah, with a nurse... I don't know!"

### Dual view of the nurse's role and competency

The issue of shared roles between doctors and nurses is an important source of anxiety for the patients we met. Many of them tended to see the nurse's role as a traditional one of assisting the doctor with various duties. Asked to describe the nurse's role, they mentioned the following activities: facilitating the doctor's tasks, making a preliminary assessment of the health problem and reporting it to the doctor, prioritizing cases to determine the order of patient consultations, taking blood samples, and performing lab tests requested by the doctor.

Participant 18-L: "So, to help the doctors do their jobs. Like, ah ... let's say when you come to the family clinic, [...] a nurse takes your blood pressure, weighs you, and so forth."

Participant 12-H: "As far as I'm concerned, the nurse is there to help the doctor. [...] Sort of between the patient and the doctor."

Participant 9-L: "Then if you have blood tests, well, she (the nurse) can do it right away."

However, other activities reflecting a broader view of the nurse's role were reported, such as providing information on health problems or prescriptions, adjusting the medication, providing follow-up for chronic disease, informing, reassuring, and treating minor conditions.

Participant 10-H: "The nurse can help in a lot of ways! Ah ... sometimes you've got a really bad cold or, ah ... an allergy, or ah [...], she could give you some advice, the names of the drugs, the ... or send you in to the doctor right away."

Participant 14-L: "She's there to make us feel more secure."

Participant 1-L: "It calms me down just to talk with the nurse instead of waiting until later to ... because when you're not sure, you rack your brains for nothing, a lot of the time."

Participant 9-L: "She can do Pap-tests; they can do Pap-tests, too, the nurses."

The issue of competence was raised by the majority of patients. Broadly speaking, participants said they were confident about the nurses' competence in most situations. However, for their own particular situation, or for certain specific tasks, they did not necessarily feel that nurses should provide treatment that was traditionally provided by their doctor, even if the nurse worked in collaboration with their doctor.

Participant 18-L: "Of course the nurses, and others, have received ... have done their studies. So, I have confidence in their qualifications."

Participant 3-L: (Talking about a drug prescription) "Oh yes, yes, yes! Yes, yes, yes! On this subject I would be very leery. Me, I would prefer that the doctor handled it."

Participant 1-L: "But, say they passed an exam ... I don't know, eh? I don't know enough about the nurse's skills, what she could have in the way of skills, and the doctor, now, ... I mean ... I know that the doctor, he can give me all the care that I need ..."

### Conditions for the successful involvement of nurses in primary care practices

Patients suggested that certain conditions must be met for the optimal involvement of nurses in primary care practices. The first condition is to establish a good information sharing and communication system between doctor and nurse. Patients expect seamless information-sharing such that information provided to a professional is available to other professionals involved in the relationship, while respecting confidentiality. However, this information sharing may be somewhat asymmetric, and in this sense, depends on the trust that must be developed, particularly with respect to the care that the nurse provides. It frequently emerged that patients expect the doctor to validate the nurse's decisions to some extent and that the communication system should serve this purpose.

Participant 1-L: "Let's say I go to see the nurse for ... I'm using the example of drugs again [...] me, if she tells me that I should stop taking this pill or start this other thing, well, I'd really like my doctor to know what's going on before the nurse does it."

Participant 3-L: "That when I go to see the doctor ... my doctor ... that he knows I've been to see the nurse ..."

Participant 1-L: (Speaking about sharing the medical chart) "Everything is confidential between the two of them"

Here again, the perceived competence of the nurse and the resulting trust appear as essential conditions including the knowledge update component.

Participant 8-L: (Talking about competence) "Someone who has kept up, who has kept abreast with the latest information, [...] Especially for ... often these types of diseases, the same things keep turning up: heart problems, diabetic complications [...], somebody who keeps informed on all that, and who has the capability to understand how it works ... oh yes! Me, I would trust that person, yeah!"

Patient 5-L: (Talking about the nurse) "But I have to trust the person!"

The roles of primary care professionals (family doctors and nurses) are perceived as having to be clearly defined so that the patient knows which services to expect from each one. This was often expressed by patients in the form of a hesitation while speaking about the role of the nurse as illustrated in the following quote.

Participant 1-L: (Talking about collaboration between a nurse and his own doctor). "Well in an office, it's... I don't know... I would see a nurse... what I mean is... for my medication, those things... it would be OK, not bad but uh... I would still like to see my family doctor to reassure me, to say, uh... really uh... in reality, my family doctor is the one who is aware of everything ... but if it's for uh... to review a prescription of something like that, I don't know... will the nurse be able to do medical acts that the, the doctor uh... can do...I don't know! If I arrive and have a pain somewhere and uh... for sure that she will not be able to give me an examination for uh..."

Among other conditions for the participation of nurses in family practices that emerge from the analyses, we should mention the nurse's proper use of health care protocols and the employment stability of health care professionals. Patients expect to keep the same nurse and to develop a long-term relationship with him/her, similar to the relationship they have with their doctor.

Participant 2-L: "But if she follows the same protocol, then... then if she has doubts, and she says "I will make, I will not make the decision, I will ask the doctor", well that's OK, trust is established."

Participant 8-L: (Talking about the long term follow-up by the same nurse). "So I don't have to start my story all over again and then uh... because my story some parts uh... are long eh!"

## Discussion

Based on this study, patients with multimorbidity seem receptive to the idea of nurses taking part in primary care practices. Through the integration of such nursing practices across the existing healthcare network, they expect greater accessibility to primary care. These results are consistent with the implementation of FMGs in Quebec, which put greater emphasis on improved accessibility to family medicine and task sharing between doctors and nurses [30,48]. In fact, the entire primary care services reform emphasizes the need to follow up and take responsibility for vulnerable patients, including individuals suffering from chronic diseases. As shown in our study, these patients require information and psychological support to better deal with their numerous chronic conditions on a daily basis. The success of preventive approaches, systematic follow-up, and continuity of care is due notably to the integration of nurses and other professionals into medical teams [49].

Nevertheless, primary care patients need reassurance that they have the freedom to choose and that they can see their family doctor when they believe that their situation requires it. Many patients in this study hold the traditional view of nursing practice, which is not representative of the optimal role for nurses to more effectively manage chronic diseases. Their conception of the nurse's role is not surprising given their experience with these professionals and the fact that the great majority of them (63%) work in hospitals [50]. At the time of the research, the involvement of primary care nurses in care for patients with chronic diseases was not significant in Quebec and varied highly among primary care organizations. However, some patients have a broader view of nurses' role in primary care and expect them to provide information, adjust their medication and provide follow-up in regard to their chronic condition, which speaks to the principles at the basis of the implementation of collaborative practices between nurses and doctors in primary care [51].

The involvement of nurses in primary care practices cannot be established without a clear definition of professional roles and fields of practice [32]. This study confirms this, but also stresses that role definition is insufficient and patients must develop confidence in the actualisation of these roles as well. Although professional medical and nursing associations have attempted to define the responsibilities of doctors and nurses in Quebec FMGs [51], collaborative procedures still vary across these organizations [52]. In addition, the nurse's role is liable to change in coming years [51] and the arrival of newly graduated nurse practitioners specialized in primary care will require further adjustments, by both the healthcare system and patients. In Quebec, practical nursing procedures are defined in a document adopted by the *Ordre des infirmières et infirmiers du Québec *(OIIQ) and the *Collège des médecins*, and as such, will guide the integration of nurses into health care teams [53].

In tangible terms, doctors and nurses working together in primary care settings with patients presenting multimorbidity as in this study, and probably other patients as well, will have to make sure that patients properly understand the role of each care provider, and that a good communication system is set up to ensure seamless information exchange between primary care professionals. Patients' comments on the importance of the quality of information sharing between professionals are consistent with literature findings on collaboration [54,55] and with the conceptual model of collaboration between professionals in healthcare organizations [56]. To successfully establish effective practices, nurses' competency must be demonstrated to, and acknowledged by clients with multiple chronic diseases. The position of the OIIQ on the requirements for university-level nursing training in primary care service [53] and the future integration of nurse practitioners in primary care [53] will probably reassure patients, particularly if the contribution of these nurses is explained. Trust in the family doctor has usually been established and the arrival of another professional can be viewed as threatening. However, it is reassuring to observe the receptiveness of the patients in our study, despite the fact that some of them had no experience with such practices involving nurses in primary care. In a study in a rural community in Canada, patients reported improvement in their psychological well-being, knowledge, and trust in managing their problems when a shared care system between family doctors and nurses was set up [57]. In another study, patients expressed their satisfaction with the collaborative care provided and reported having a greater feeling of control over their situation [58].

Results of this study indicate the need for better planning of the arrival of primary care nurses, as well as a concerted effort to support the change process [51]. As Bailey [35] concludes, it takes more than just nurses and family doctors working in a shared practice to produce collaborative practices. No doubt the presence of nurses in primary care is a useful addition from the standpoint of the decision-makers and professionals concerned. However, the main users are the patients, and in the end, they are the ones to judge whether or not the involvement of nurses in primary care practices is successful.

This study does have its limitations such as a small sample size. Furthermore, it is limited to a single, peripheral geographic region, deliberately addressing a particular group that is affected by chronic diseases and has considerable experience using primary care services. Nonetheless, we believe that the results are transferable to other patients typically seen in primary care given the diversity of the sample in terms of chronic diseases and types of settings, in-depth description of perceptions and variation among these perceptions, and data saturation. However, transferability to other settings or primary care systems should be done with caution. One should also take into account regional differences in regard to context, such as the absence of primary care nurse practitioners in Quebec. All participants in this study received follow-up care for several years; patients with a shorter follow-up period may have different perceptions. Finally, findings could have been enriched by the addition of a second interview and the exploration of the nursing practice primary care context to deepen the understanding and to clarify participants' perspectives.

Various triangulation methods were used to ensure research rigor and results reliability: triangulation of analyses, researchers and disciplines (family medicine, nursing, social work). This combination of triangulations improves rigour by taking into account a variety of viewpoints and ensuring that empirical data and descriptions are well matched [43,59,60]. However, the results are mainly descriptive and less interpretative of the experience of patients with multimorbidity regarding nursing care in primary care settings. Further qualitative research would help gain a better understanding of this experience. One other strength of this study is the focus on the patient's viewpoint, a factor that has received little attention to date. The timing of the research is also highly appropriate, as primary care services are undergoing a major reform particularly in Quebec. Future research might consider replicating the study in other settings to assess the acceptability of nurses in primary care teams.

## Conclusion

Patients suffering from multiple chronic diseases are receptive to the involvement of nurses in primary care practices. They expect a greater accessibility to services, for themselves and for new patients taken on by the primary care team as a result. However, they expect a clear definition of each profession's (doctors and nurses) roles and fields of practice.

## Competing interests

The authors declare that they have no competing interests.

## Authors' contributions

MF participated in the conception and design of the study, supervised data collection and analysis and drafted the manuscript. CH participated in the study design and critical review of the manuscript. FG participated in the study design, data analysis and helped draft the manuscript. ALN and DM participated in the data analysis and critical review of the manuscript. HS critically reviewed and helped draft the manuscript. All authors gave their final approval of the version of the manuscript submitted for publication.

## Pre-publication history

The pre-publication history for this paper can be accessed here:

http://www.biomedcentral.com/1471-2296/11/84/prepub
